# Differences in pregnancy and perinatal outcomes among symptomatic versus asymptomatic COVID-19-infected pregnant women: a systematic review and meta-analysis

**DOI:** 10.1186/s12884-021-04250-1

**Published:** 2021-12-01

**Authors:** Durray Shahwar A. Khan, La-Raib Hamid, Anna Ali, Rehana A. Salam, Nadeem Zuberi, Zohra S. Lassi, Jai K. Das

**Affiliations:** 1 Department of Pediatrics, Aga Khan University, Karachi, 74800 Pakistan; 2 Robinson Research Institute, University of Adelaide, Adelaide, 5005 Australia; 3 Department of Obstetrics and Gynaecology, Aga Khan University, Karachi, 74800 Pakistan

**Keywords:** Coronavirus, SARS-CoV-2, COVID-19, Pregnancy, Pregnant women, Clinical presentation

## Abstract

**Background:**

There is dearth of information on COVID-19’s impact on pregnant women. However, literature reported trends of COVID-19 differ, depending on the presence of clinical features upon presentation.

**Objective:**

This systematic review aimed to assess differences in risk factors, management, complications, and pregnancy and perinatal outcomes in symptomatic vs. asymptomatic pregnant women with confirmed SARS-CoV-2 infection.

**Methods:**

A search was run on electronic databases to identify studies reporting COVID-19 in pregnancy. Meta-analysis was performed and odds ratios and mean difference with 95% confidence intervals were calculated using Review Manager 5.4. Review Prospero registration number CRD42020204662.

**Results:**

We included ten articles reporting data from 3158 pregnancies; with 1900 symptomatic and 1258 asymptomatic pregnant women. There was no significant difference in the mean age, gestational age, and body mass index between the two groups. The meta-analysis suggested that pregnant women who were obese (OR:1.37;95%CI:1.15 to 1.62), hypertensive (OR:2.07;95%CI:1.38 to 3.10) or had a respiratory disorder (OR:1.64;95%CI:1.25 to 2.16), were more likely to be symptomatic when infected with SARS-CoV-2. Pregnant women with Black (OR:1.48;95%CI:1.19 to 1.85) or Asian (OR:1.64;95%CI:1.23 to 2.18) ethnicity were more likely to be symptomatic while those with White ethnicity (OR:0.63;95%CI:0.52 to 0.76) were more likely to be asymptomatic. Cesarean-section delivery (OR:1.40;95%CI:1.17 to 1.67) was more likely amongst symptomatic pregnant women. The mean birthweight(g) (MD:240.51;95%CI:188.42 to 293.51), was significantly lower, while the odds of low birthweight (OR:1.85;95%CI:1.06 to 3.24) and preterm birth (< 37 weeks) (OR:2.10;95%CI:1.04 to 4.23) was higher amongst symptomatic pregnant women. Symptomatic pregnant women had a greater requirement for maternal ICU admission (OR:13.25;95%CI:5.60 to 31.34) and mechanical ventilation (OR:15.56;95%CI:2.96 to 81.70) while their neonates had a higher likelihood for Neonatal Intensive Care Unit admission (OR:1.96;95%CI:1.59 to 2.43). The management strategies in the included studies were poorly discussed, hence could not be analyzed.

**Conclusion:**

The evidence suggests that the presence of risk factors (co-morbidities and ethnicity) increased the likelihood of pregnant women being symptomatic. Higher odds of complications were also observed amongst symptomatic pregnant women. However, more adequately conducted studies with adjusted analysis and parallel comparison groups are required to reach conclusive findings.

**Supplementary Information:**

The online version contains supplementary material available at 10.1186/s12884-021-04250-1.

## Introduction

In December 2019, a rising number of cases of ‘pneumonia of unknown etiology’ emerged in Wuhan, Hubei Province, China. Consequently, the severe acute respiratory syndrome coronavirus 2 (SARS-CoV-2) was identified as a novel coronavirus in this outbreak [1]. The mode of transmission of this virus is mainly via respiratory droplets, secretions and direct contact [2]. With the rapid spread of the disease worldwide, the novel coronavirus disease (COVID-19) was declared a public health emergency of international concern by the World Health Organization (WHO) [3]. By March 2020, it was declared a pandemic [4], and globally, as of October 21, 2020, a total of 40.2 million cases have been confirmed, with more than 1.1 million deaths [5].

The implications of COVID-19 amongst the vulnerable population, particularly pregnant women, is of utmost concern, as alterations in cell-mediated immunity in pregnancy may increase the susceptibility to intracellular pathogens such as viruses [6]. The anatomical and physiological changes occurring during pregnancy such as the rising transverse diameter of the thorax, elevation of the diaphragm, alterations in lung volumes, and vasodilation with subsequent mucosal edema may lessen the maternal tolerance to hypoxia and later, concur adverse outcomes [7]. Moreover, it has been observed that during pandemics, an increase in severity of the disease expression is recorded amongst the pregnant population. In the 1918 Influenza pandemic, maternal mortality was observed to be 27% amongst those affected by the disease [8]. The clinical outcomes among pregnant women from the previous two coronavirus diseases, the SARS-CoV and Middle East respiratory syndrome coronavirus (MERS- CoV), were less encouraging compared to the non-pregnant women [9–11].

Due to previously noted maternal and neonatal complications with SARs and MERS; the concern of increased risk of maternal and fetal complications with COVID-19 has been high and since the start of the pandemic, multiple studies have focused on the clinical features and outcomes of pregnant women with COVID-19 [12, 13]. New data on pregnant women affected by COVID-19 is emerging with each passing day, but it is imperative to evaluate the differences in the risk factors, management, and pregnancy and perinatal outcomes between pregnant women having laboratory-confirmed coronavirus with varying clinical presentation. Therefore, this systematic review aims to assess the difference, if any, in the risk factors (co-morbidities and ethnicity), management, and pregnancy and perinatal outcomes between symptomatic and asymptomatic COVID-19 confirmed pregnant women. This shall enable healthcare professionals to plan out management for any obstetric patient affected by COVID-19 infection and take timely decisions.

## Methodology

This systematic review has been registered with the International Prospective Register of Systematic Reviews (PROSPERO) database under the Registration number CRD42020204662. It follows the guidelines recommended by the Preferred Reporting Items for Systematic Reviews and Meta-Analyses (PRISMA) [14] (Supplementary Table 1).

We conducted an electronic search using PubMed, Embase, the WHO COVID-19 Database, and Google Scholar until February 25, 2021. Preprint databases, namely MedRxiv and BioRxiv were also explored using keywords. The following terms and their variants were used in our search strategy: “Coronavirus” OR “COVID-19” OR “SARS-CoV-2” AND “Pregnancy” OR “Pregnant women”. The full search strategy is attached in supplementary Table 2.

We included observational studies (cohort, case-control, cross-sectional, and case series, but excluded case reports) including consecutive patients and with a comparison of symptomatic and asymptomatic confirmed cases of pregnant women with COVID-19. The outcomes included risk factors, management, pregnancy outcomes, and perinatal outcomes. We excluded studies that only reported the number of symptomatic and asymptomatic pregnant women with COVID-19 without reporting outcomes separately for each group, studies that grouped asymptomatic with mild COVID-19 cases and compared it to moderate and severe COVID-19. Studies that included data from similar settings and during the same time period were assessed for data overlap. Where information was unclear, authors were contacted to confirm the center of data collection and their time period to ensure there was no repetition of data. Identified overlapping papers were further assessed and the studies with inclusion of more variables, bigger sample size, and better quality of assessment were chosen as shown in supplementary Table 3. We did not apply any language restrictions while screening articles.

Two reviewers (DSK and LH) independently screened titles and abstracts, full texts were then reviewed for relevant data and examined for fulfilling the inclusion criteria. Any discrepancies were resolved by discussion with the senior reviewer (ZSL). Two review authors independently assessed the risk of bias for each study using the National Heart, Lung, and Blood Institute (NHLBI) quality assessment tool for cohort and case-series studies [15]. This tool helps evaluate the internal validity of a study, hence ensuring that the results are truly due to the exposure being evaluated. Two reviewers (DSK and LH) independently extracted data from relevant articles on the following variables: author’s name, study design, location (center, city, and country of data collection), time period of data collection, sample size, management (intensive care unit (ICU) management), pregnancy outcomes (mode of delivery) and perinatal outcomes (e.g. preterm birth, mean birthweight, low birth weight (LBW), APGAR scores at 1 and 5 min, neonatal intensive care unit (NICU) admission, perinatal mortality (including stillbirths and neonatal death), and SARS-CoV-2 infection in neonates, etc.).

The analysis was carried out using Review Manager (RevMan) version 5.4 [16]. Continuous data were reported as mean difference (MD) with 95% confidence interval (CI) whereas dichotomous data were reported using odds ratio (OR) with 95% CI. Heterogeneity between the studies was explored using the *p*-value of chi-square and I^2^ statistic and a random effect model was used.

## Results

**Fig. 1:**
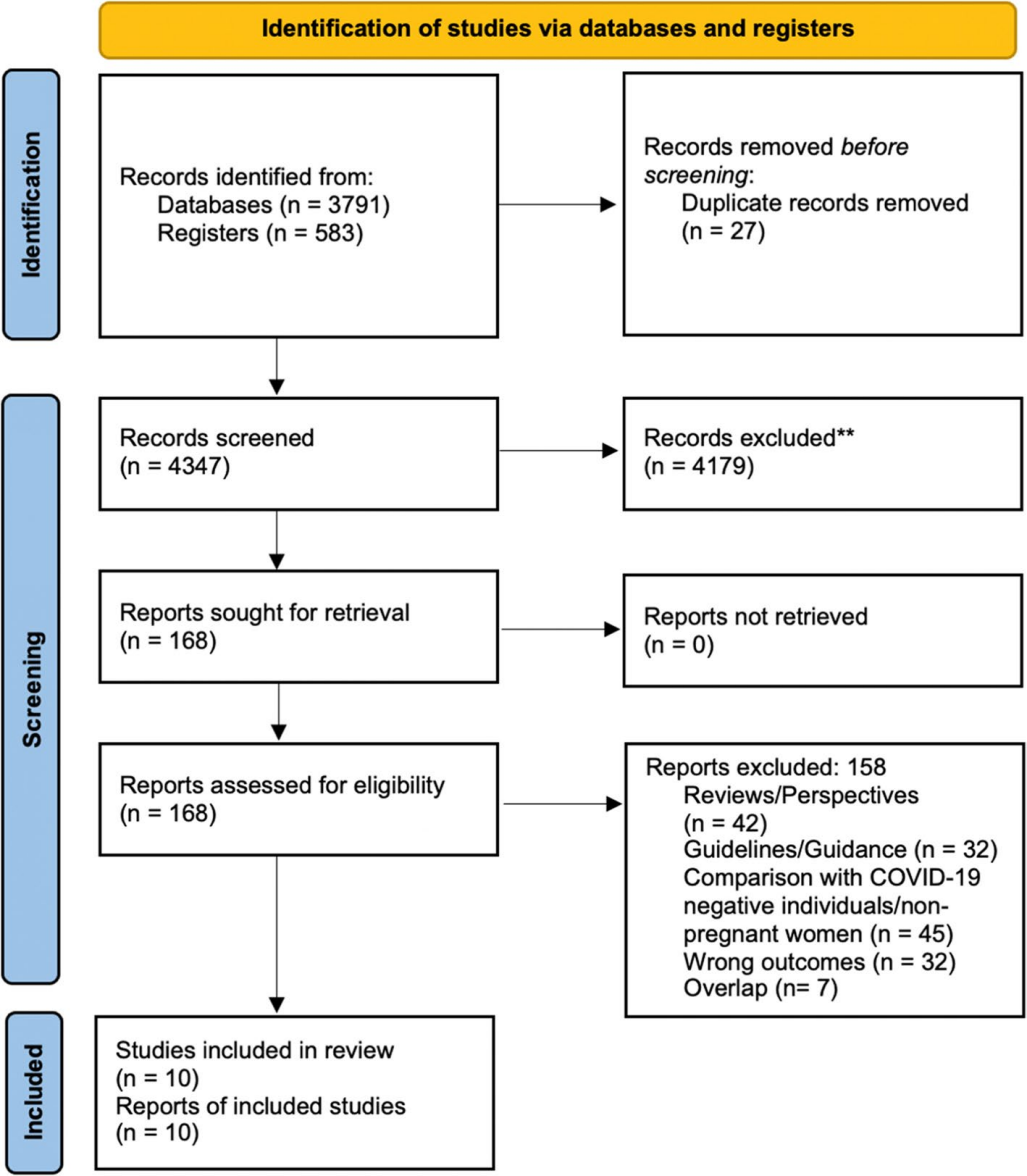
PRISMA Flow Diagram

There were a total of 4347 articles identified after running the search strategy on all electronic databases. After screening titles and abstracts, 4179 were excluded. Of the 168 studies retrieved for full-text review, 158 studies were excluded, of which 42 were authors’ perspectives or reviews, 32 were guidelines or guidance papers based on other coronavirus strains, 45 studies compared SARS-CoV-2 infected pregnant women with non-infected individuals or SARS-CoV-2 infected non-pregnant women, 32 studies did not report any outcome of interest and seven studies were excluded due to overlap as they were conducted at a similar center during the same time period. A total of ten studies with 3158 participants that met the eligibility were included in this review (Fig. 1).

All the ten studies were observational; with six cohort studies (*n* = 3032) [17–22]; three case-series (*n* = 36) [23–25], with only one having a sample size greater than 10 [24] and one case-control study (*n* = 45) [26]. The data from included cases were collected between December 2019 to November 2020 and all manuscripts were published between the years 2020 and 2021. Three studies were conducted in Wuhan, China [23–25], three in different states across the United States of America [20–22], one in London, United Kingdom [19], one in Muscat, Oman [18], one in Hamadan province, Iran [26] and one was a multi-country study that included cases from 73 centers of 22 countries [17]. The countries included were Argentina, Australia, Belgium, Brazil, Colombia, Czech Republic, Finland, Germany, Greece, Israel, Italy, North Macedonia, Peru, Portugal, Republic of Kosovo, Romania, Russia, Serbia, Slovenia, Spain, Turkey, and United States. Five of the included studies were from a single-center [18, 22–25] whereas four were multicenter studies [19–21, 26], and one was a multicountry multicenter study [17]. The quality assessment for cohort studies revealed all studies to have described their objectives and study subjects well. They all had individuals recruited from the same populations and had uniform criteria for measuring exposure. However, none of the studies assessed a varying level of exposure, nor was the exposure measured at different time intervals as there was no follow-up. The quality assessment for the case series revealed all studies to have adequate individual case descriptions with clear objectives and methods of analysis except one study [25]. The quality assessment for a single study with a case-control study design specified objective and study subjects with controls recruited from the same population. Quality assessment for each study is depicted in Supplementary Tables 4, 5, and 6.

The number of enrolled individuals in each study ranged from seven to 1219. Six studies had a sample size of less than 100 participants [18, 22–26], two studies had a sample between 100 to 500 participants [17, 20], and two studies had a sample size greater than 1000 but less than 1300 participants [19, 21]. Eight of the studies used RT-PCR as the method of confirming SARS-CoV-2 infection [17, 18, 20, 22–26], one used molecular or antigen test [21], and one study had not specified the testing technique/s used [19]. The characteristics of included studies are reported in Table 1 and their methodological quality in Supplementary Tables 4, 5, and 6.

**Table 1:** Characteristics of included studies

Study and year	Study design	Country and time period	Setting	Total number	Demographics		Past Medical History
					Symptomatic	asymptomatic	Symptomatic
Dongmei Cao 2020 [23]	Case-series	Wuhan, China Jan 23rd to Feb 23rd 2020	Maternal and Child health hospital	Total = 10 Symp = 7 Asymp = 3	Mean age:30.6 Mean Gest. Age: 37 + 6	Mean Age: 29.7 Mean Gest. Age: 37 + 5	Hypothyroid = 1 Anemia = 1
Ensiyeh Jenabi, 2020 [26]	Case-control	Hamadan Province, Iran Sept 1 to Nov 15, 2020	Hospitals of Hamadan Province located in West Iran	Total = 90 Symp = 45 Asymp = 45	Mean age = 29.47 Mean Gestational age = 37.13	Mean age = 28.78 Mean Gestational age = 37.62	Comorbidity disease = 3
Gabrielle Saccone 2020 [17]	Retrospective cohort	22 different countries (Argentina, Australia, Belgium, Brazil, Colombia, Czech Republic, Finland, Germany, Greece, Israel, Italy, North Macedonia, Peru, Portugal, Republic of Kosovo, Romania, Russia, Serbia, Slovenia, Spain, Turkey, and United States) February 1, 2020 and April 30, 2020	Various hospitals	Total = 388 Symp = 294 Asymp = 94	N/A	N/A	N/A
Jayasree Santhosh2020 [18]	Cohort study	Muscat, Oman March 24 to July 31, 2020	The Royal Hospital	Total = 60 Symp = 44 Asymp = 16	N/A	N/A	Hypertension = 1 Diabetes = 2 GDM = 12 Hematologic disease = 7
Nicola Vousden2021 [19]	Prospective Cohort	United Kingdom March 1 to August 31, 2020	194 hospitals vis UK Obstetric Surveillance System (UKOSS)	Total = 1148 Symp = 722 Asymp = 426	Race-ethnicity: White = 318 Asian = 210 Black = 122 Chinese = 8 Other = 36 Mixed = 15 Current smoking = 42	Race-ethnicity: White = 276 Asian = 84 Black = 33 Chinese = 4 Other = 16 Mixed = 5 Current smoking = 57	Asthma = 49 Hypertension = 24 Cardiac disease = 13 Diabetes = 22
Sourabh Verma, 2020 [20]	Cohort study	New York, USA March 1 to May 10, 2020	New York University (NYU) Langone Health system hospitals (NYU Tisch, NYU Brooklyn, NYU Winthrop) and Bellevue Hospital Center	Total = 149 Symp = 89 Asymp = 60	Mean age = 32 BMI (> = 30) = 38	Mean age = 30.4 BMI (> = 30) = 24	Asthma = 6 Diabetes = 2 Gestational Diabetes = 5 Gestational hypertensio*n* = 12
Torri Metz 2021 [21]	Cohort study	14 states USA March 1st to July 31st, 2020	33 National Institute of Child Health and Human Development (NICHD) Maternal-Fetal Medicine Units (MFMU) sites	Total = 1219 Symp = 640 Asymp = 579	Mean Age = 30 BMI (> = 30) = 328 Race-ethnicity: Black = 152 White = 101 Hispanic = 327 Smoked = 23	Mean Age = 28 BMI (> = 30) = 561 Race-ethnicity: Black = 123 White = 80 Hispanic = 324 Smoked = 27	Asthma or COPD = 114 Pregestational Diabetes = 32 Hypertension = 51 Cardiovascular disease = 11 Chronic renal disease = 3 Chronic liver disease = 5 Thyroid disease = 32 Neurocognitive disorder = 25 Neuromuscular disorder = 3 Seizure disorder = 10 Inflammatory bowel disease = 3
Viktoriya London 2020 [22]	Retrospective Cohort study	Brooklyn, NY, United States of America From March 15th to April 10th 2020	Maimonades Medical Centre	Total = 68 Symp = 46 Asymp = 22	Mean age = 30 BMI = 31 Gestational age = all in 3rd trimester (except 17wks (n = 1), 25 wks (n = 1) and 26 wks (n = 1) Nulliparity = 12	Mean age = 30.5 BMI = 31.6 Gestational age = all third trimester Nulliparity = 8	Comorbidity = 15/46 Preeclampsia = 2
Xiaolin Hu 2020 [25]	Case series	Wuhan, China Jan 20th to Feb 20th	Tongji Medical college	Total = 7 Symp = 6 Asymp = 1	32.7 + − 1.6 Gestational Age = 39 + 1	33 Gestational age = 38 + 2	N/A
Xiaoqing Wu 2020 [24]	Case series	Wuhan, China Dec 31st to March 17th 2020	Central Hospital of Wuhan	Total 19 Symp = 7 Asymp = 12	Mean age = 28.3	Mean age = 29.1	Hepatitis B = 1 PIH = 0 Hypothyroid = 0

Study and year	Past Medical History	Management		Delivery and Neonatal Characteristics and Outcomes		Complications	
	Asymptomatic	Symptomatic	Asymptomatic	Symptomatic	Asymptomatic	Symptomatic	Asymptomatic
Dongmei Cao 2020 [23]	Comorbid = none	N/A	N/A	Vaginal birth = 1 Total CD = 6 Elective CD = 4 Intrapartum = 2 Livebirths = 7/7 Birthweight = 3112.14 Neonatal asphyxia = 0 Preterm birth = 2 1 min Apgar score = 8.7 5 min Apgar score = 10 Neonatal death = 0 Neonatal PCR = all negative	Vaginal birth = 1 Total CD = 2 Elective CD = 2 Intrapartum CD = 0 Livebirths = 4/3 (single pair of twins) Birthweight = 3215 Neonatal asphyxia = 0 Preterm birth =1(twins) 1 min Apgar score = 8.3 5 min Apgar score = 10 Neonatal death = 0 Neonatal PCR = all negative	GDM = 1 Pre-eclampsia = 3 Placental abruption = 1 Fetal distress = 1 PROM = 2 Preterm = 2 Neonatal death = 0	GDM = 0 Pre-eclampsia = 0 Placental abruption = 0 Fetal distress = 1 PROM =2 Preterm- = 1 Neonatal death = 0
Ensiyeh Jenabi, 2020 [26]	Comorbidity disease = 19	N/A	N/A	Preterm labour = 12 C-section = 27 LBW = 12 Neonatal death = 2	Preterm labour = 6 C-section = 12 LBW = 4 Neonatal death = 1	Preeclampsia = 11	Preeclampsia = 4
Gabrielle Saccone 2020 [17]	N/A	N/A	N/A	C-section = 100/177 Mean birthweight = 2821 ± 846 IUGR = 9/189 Admission to NICU = 50/177 LBW = 43/177 Neonatal Death = 5/177	C-section = 36/74 Mean birthweight = 3149 ± 496 IUGR = 1/77 Admission to NICU = 19/74 LBW = 9/74 Neonatal death = 0/74	Maternal ICU admission = 42/294 Mechanical ventilation = 35/294	Maternal ICU admission = 1/94 Mechanical ventilation = 1/94
Jayasree Santhosh2020 [18]	Hypertension = 2 Diabetes = 3 GDM = 3 Hematologic disease = 1	N/A	N/A	Normal Vaginal = 19 Instrumental delivery = 1 Elective C-section = 4 Emergency C-section = 12 Preterm birth = 12 Stillbirth = 1 LBW = 9	Normal Vaginal = 5 Instrumental delivery = 3 Elective C-section = 2 Emergency C-section = 3 Preterm birth = 6 Stillbirth = 0 LBW = 6	Preterm PROM = 1 Miscarriage = 1	Preterm PROM = 0 Miscarriage = 0
Nicola Vousden2021 [19]	Asthma = 28 Hypertension = 2 Cardiac disease = 8 Diabetes = 6	Oseltamivir = 11 Steroids = 120	N/A	C-section = 314 Vaginal = 308 Preterm birth (< 37 weeks) = 114 Stillbirth = 5 Live birth = 627 NICU admission = 121 Neonatal death = 2	C-section = 153 Vaginal = 228 Stillbirth = 4 Live birth = 381 NICU admission = 35 Neonatal death = 2	Required critical care = 63 COVID-19 pneumonia = 173 Pre-eclampsia = 15 Death = 8	N/A
Sourabh Verma, 2020 [20]	Asthma = 6 Diabetes = 2 Gestational Diabetes = 5 Gestational hypertension = 5	N/A	N/A	Alive = 89 C-section = 23 Preterm birth (< 37 weeks) = 14 NICU admission = 17 Neonatal death = 1 Positive neonatal RT-PCR = 1/66	Alive = 60 C-sectio*n* = 13 Preterm birth (< 37 weeks) = 2 NICU admission = 1 Neonatal death = 0 Positive neonatal RT-PCR = 0/21	ICU admission = 8	ICU admission = 9
Torri Metz 2021 [21]	Asthma or COPD = 51 Pregestational Diabetes = 17 Hypertension = 29 Cardiovascular disease = 3 Chronic renal disease = 0 Chronic liver disease = 1 Thyroid disease = 22 Neurocognitive disorder = 10 Neuromuscular disorder = 1 Seizure disorder = 4 Inflammatory bowel disease = 0	N/A	N/A	C-section = 253 Preterm birth (< 37 weeks) = 135 Live births = 626 SGA = 70 Birthweight = 3.5 NICU admission = 163	C-section = 197 Preterm birth (less than 37 weeks) = 69 Live births = 570 SGA = 56 Birthweight = 3.2 NICU admission = 91	Maternal death = 6 Venous thromboembolism = 9 Maternal ICU admission = 56 Postpartum hemorrhage = 66 Hypertensive disorder of pregnancy = 176 Neonatal death = 3	Maternal death = 0 Venous thromboembolism = 0 Maternal ICU admissio*n* = 3 Postpartum hemorrhage = 42 Hypertensive disorder of pregnancy = 109 Neonatal death = 2
Viktoriya London 2020 [22]	Comorbidity = 4/22 Preeclampsia = 1	Hydroxychloroquine and azithromycin = 16 Respiratory support = 12 [nasal cannula (*n* = 7); nonrebreather mask (n = 3); Hi-flow nasal cannula (n = 1); Mechanical ventilation (n = 1)	Hydroxychloroquine and azithromycin = 0 Respiratory support = 0	C-section = 16 Preterm < 37 wks = 9 Preterm < 34 wks = 3 Preterm due to: maternal respiratory distress (n = 7); decreased fetal movements (n = 1)	C-section = 6 Preterm < 37 wks = 0 Preterm < 34 wks = 0	N/A	N/A
Xiaolin Hu 2020 [25]	N/A	Anti-viral = 1	Anti-viral = 0	SVD = 1 C-section = 5 Birthweight = 3356.67 1 min Apgar = 8 5 min Apgar = 9 COVID RT-PCR = 1 positive	SVD = 0 C-section = 1 Birthweight = 3180 1 min APGAR = 7 5 min APGAR = 8 Covid RT-PCR = all negative	Liver dysfunction	No complications
Xiaoqing Wu 2020 [24]	Hepatitis B = 1 PIH = 3 Hypothyroid = 1	N/A	N/A	N/A	N/A	PROM = 0 Fetal intrauterine hypoxia = 0	PROM = 1 Fetal intrauterine hypoxia = 1

*Asymp* Asymptomatic pregnant women, *BMI* Body Mass Index, *C-section* Cesarean section, *Chr* Chronic, *GDM* Gestational Diabetes Mellitus, *ICU* Intensive Care Unit, *IUGR* Intrauterine Growth Retardation, *LBW* Low Birth Weight, *NICU* Neonatal Intensive Care Unit, *PCR* Polymerase Chain Reaction, *PIH* Pregnancy Induced Hypertension, *PROM* Premature Rupture of Membranes, *SVD* Spontaneous vaginal delivery, *Symp* Symptomatic pregnant women, *Wks* weeks

All of the participants included in this review were SARS-CoV-2 infected pregnant women with 1900 symptomatic individuals and 1258 asymptomatic individuals. The meta-analysis did not find any significant difference in mean age, gestation age, and BMI between symptomatic and asymptomatic COVID-19 affected pregnant women as reported in Table 2. There was no statistically significant difference between symptomatic and asymptomatic pregnant women who were nulliparous. The comparison according to different ethnic groups revealed the odds of being symptomatic was greater amongst Black (OR 1.48; 95% CI: 1.19 to 1.85; 2 studies, 2367 participants) and Asian (OR 1.64; 95% CI: 1.23 to 2.18; 1 study, 1148 participants) ethnicities. However, individuals from the White ethnicity were more likely to be asymptomatic (OR 0.63; 95% CI: 0.52 to 0.76; 2 studies, 2367 participants) (Table 2).

**Table 2:** Comparison of Symptomatic and Asymptomatic pregnant women with COVID-19: summary estimates

Outcomes	Odds Ratio/Mean Difference (95% CI)	No of studies; No of participants	Heterogeneity Chi^**2**^ P-value; I^**2**^
**Demographics**			
Mean age (years)	1.01 [−0.10 to 2.12]	5; 324	0.89; 0%
Advanced maternal age	1.62 [0.05 to 51.11]	1; 10	N/A
Gestational age on admission	−0.56 [−1.67 to 0.56]	2; 100	0.34; 0%
Mean BMI	0.60 [−6.39 to 5.19]	1; 68	N/A
Nulliparity	0.62 [0.21 to 1.84]	1; 68	N/A
Hispanic	0.82 [0.66 to 1.03]	1; 1219	N/A
Asian	1.64 [1.23 to 2.18]	1; 1148	N/A
Black	1.48 [1.19 to 1.85]	2; 2367	0.003; 89%
White	0.63 [0.52 to 0.76]	2; 2367	0.00001; 96%
**Past medical history**			
Smoking	0.50 [0.36 to 0.71]	2; 2367	0.07; 69%
Comorbidity	0.53 [0.05 to 5.84]	3; 165	0.003; 82%
Obesity	1.37 [1.15 to 1.62]	3; 2516	0.83; 0%
Hypertension	2.07 [1.38 to 3.10]	3; 2427	0.03; 72%
Cardiovascular disease	1.53 [0.75 to 3.12]	2; 2367	0.11; 60%
Diabetes mellitus	1.25 [0.57 to 2.77]	4; 2576	0.12; 48%
Hypothyroidism	0.39 [0.02 to 9.13]	1; 23	N/A
Respiratory disease/Asthma	1.64 [1.25 to 2.16]	3; 2516	0.01; 78%
**Pregnancy complications**			
Intrauterine growth retardation	3.80 [0.47 to 30.52]	1; 266	N/A
GDM	1.06 [0.73 to 1.54]	3; 1357	0.64; 0%
Preeclampsia/PIH	1.84 [1.01 to 3.38]	5; 1465	0.53; 0%
PROM	0.39 [0.02 to 9.13]	1; 23	N/A
**Complications**			
Maternal ICU admission	13.25 [5.60 to 31.34]	3; 1756	0.65; 0%
Mechanical ventilation	15.56 [2.96 to 81.70]	2; 443	0.68; 0%
Maternal Death	11.87 [0.67 to 211.22]	1; 1219	N/A
**Pregnancy and neonatal outcomes**			
C-section delivery	1.40 [1.17 to 1.67]	8; 2982	0.36; 8%
Vaginal delivery	0.74 [0.43 to 1.27]	3; 1218	0.29; 18%
Preterm birth < 37 weeks	2.10 [1.04 to 4.23]	6; 1760	0.03; 59%
Preterm birth < 34 weeks	4.34 [0.52 to 36.14]	2; 115	0.87; 0%
Neonatal Asphyxia	N/A	1; 11	N/A
Mean birthweight (g)	240.96 [188.42 to 293.51]	4; 1488	< 0.00001; 96%
Low birth weight	1.85 [1.06 to 3.24]	3; 401	0.03; 71%
Stillbirths	1.05 [0.37 to 2.97]	3; 1345	0.74; 0%
Mean APGAR score at 1 min	0.36 [−0.19 to 0.91]	2; 18	N/A
Mean APGAR score at 5 min	N/A	2; 18	N/A
NICU admission	1.96 [1.59 to 2.43]	4; 2637	0.06; 60%
Neonatal death	1.57 [0.59 to 4.17]	6;2754	0.82; 0%
Neonatal COVID-19 infection	0.91 [0.08 to 10.31]	3; 98	0.94; 0%

With regard to past medical history (existing co-morbidities); hypertensive pregnant women with COVID-19 were more likely to be symptomatic (OR 2.07; 95% CI 1.38 to 3.10; 3 studies, 2427 participants) as were pregnant women with respiratory disease (OR 1.64; 95% CI: 1.25 to 2.16; 3 studies, 2516 participants). However, there was no statistical difference in diabetic and hypothyroid pregnant women or pregnant women with chronic cardiac disease for being symptomatic or asymptomatic. Obese pregnant women with COVID-19 had greater odds of being symptomatic (OR 1.37; 95% CI 1.15 to 1.62; 3 studies, 2516 participants). Our meta-analysis also reports pregnant women who smoked to have lower odds of being symptomatic (OR 0.50; 95% CI: 0.36 to 0.71; 2 studies, 2367 participants).

**Fig. 2:**
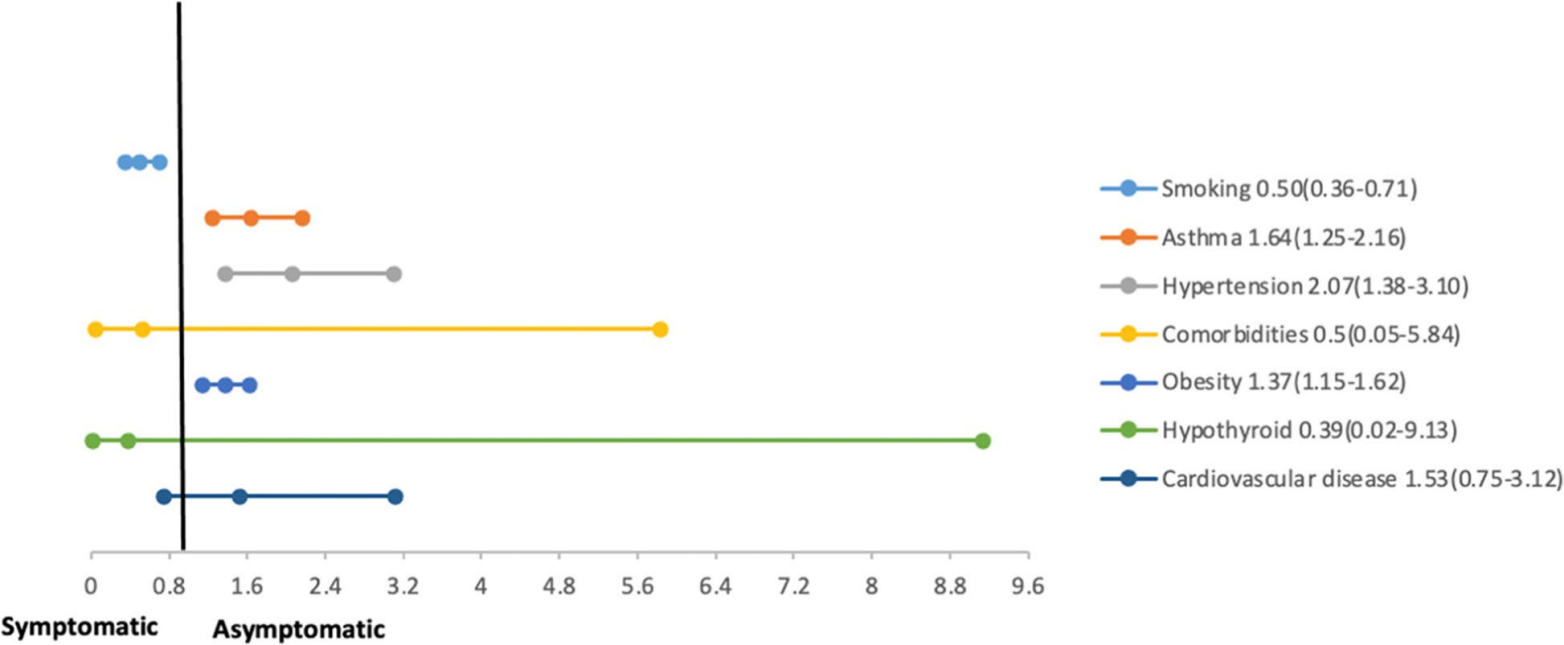
Past medical history among symptomatic and asymptomatic pregnant women with COVID-19

There was no difference between gestational diabetes mellitus (GDM), premature rupture of membranes, or intrauterine growth retardation (IUGR) with a predilection for either being symptomatic or asymptomatic amongst pregnant women with COVID-19. However, the odds of being symptomatic were higher in pregnant women with preeclampsia or pregnancy-induced hypertension (OR 1.84; 95% CI 1.01 to 3.38; 5 studies, 1465 participants). A summary of forest plots has been presented in Fig. 2 and individual forest plots can be accessed in the supplementary file as Supplementary Figs. 1 to 8.

**Fig. 3:**
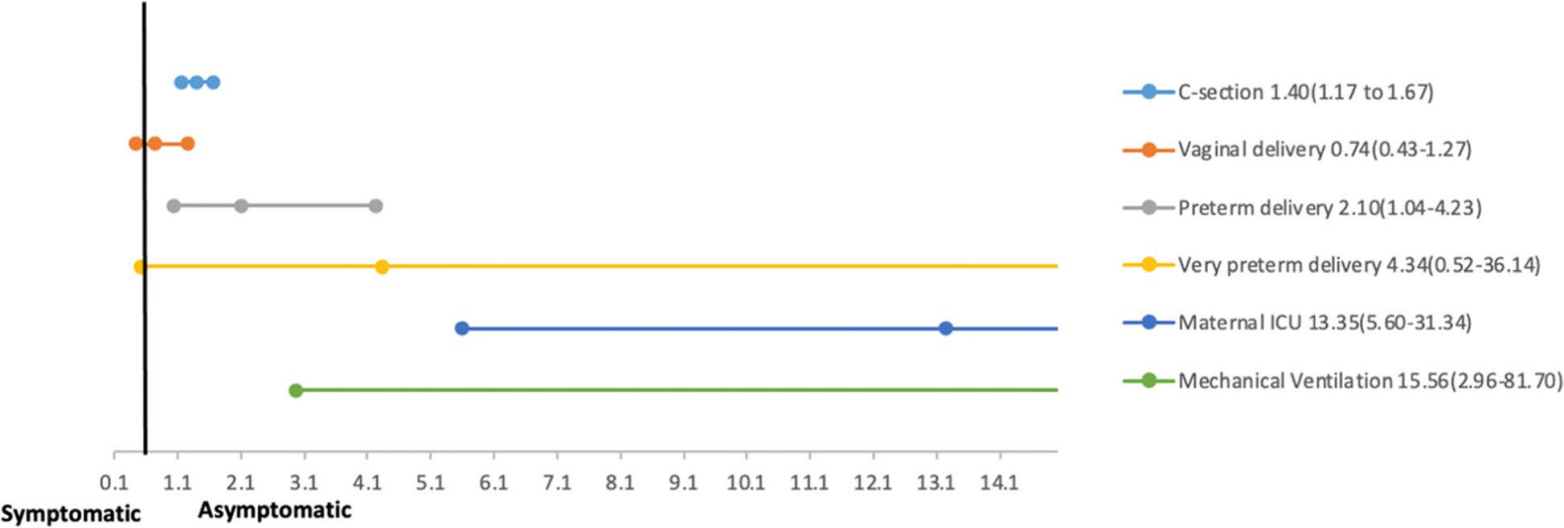
Pregnancy and perinatal Outcomes among symptomatic and asymptomatic pregnant women with COVID-19

The odds were greater for symptomatic pregnant women with COVID-19 to deliver via a cesarean section (OR 1.40; 95% CI 1.17 to 1.67; 8 studies, 2982 participants), while there was no difference in the odds of vaginal delivery across the two groups. Our meta-analysis reported mean birthweight to be lower in neonates of symptomatic pregnant women with COVID-19 (MD 240.96 g; 95% CI 188.42 to 293.51; 4 studies, 1488 participants). Similarly, the odds of having an LBW newborn (OR 1.85; 95% CI 1.06 to 3.24; 3 study, 401 participants) and preterm birth less than 37 weeks (OR 2.10; 95% CI 1.04 to 4.23; 6 studies, 1760 participants) was higher amongst symptomatic women. There was no difference in mean APGAR score at 1-min, neonatal SARS-CoV-2 infection, and neonatal death between either group. Neonatal Intensive Care Unit (NICU) admissions were more likely amongst neonates of symptomatic mothers with COVID-19 (OR 1.96; 95% CI 1.59 to 2.43; 4 studies, 2637 participants). The odds of maternal ICU admission (OR 13.25; 95% CI 5.60 to 31.34; 3 studies, 1756 participants) and mechanical ventilation requirement (OR 15.56; 95% CI 2.96 to 81.70, 2 studies, 443 participants) were also higher in symptomatic pregnant women. A summary of forest plots has been presented in Fig. 3 and individual forest plots can be accessed in the supplementary file as Supplementary Figs. 9 to 15. The management strategies could not be compared between the symptomatic and asymptomatic pregnant women infected with COVID-19 due to lack of available data.

## Discussion

Since the start of the COVID-19 pandemic, healthcare workers have been at the forefront to gather enough data in order to understand this disease better. This review primarily focused on pregnant women with COVID-19 and summarizes the differences in their risk factors, management along with their pregnancy and neonatal outcomes between symptomatic and asymptomatic pregnant women. To the best of our knowledge, this meta-analysis is the first of its kind to analyse the difference in pregnancy and perinatal outcomes that could potentially be affected, depending on the severity of COVID-19 disease.

In the present meta-analysis, we found that being symptomatic varied across different ethnicities with Black and Asian pregnant women having a higher likelihood of being symptomatic while pregnant women of White ethnicity had a higher likelihood of being asymptomatic. We also found that obese, hypertensive, and asthmatic pregnant women with COVID-19 have a higher likelihood of being symptomatic. Symptomatic pregnant women with COVID-19 had higher chances of delivering via cesarean-section while asymptomatic pregnant women with COVID-19 had a higher chance of having a vaginal delivery. The mean birthweight was lower among neonates of symptomatic pregnant women and the odds of LBW was also higher among symptomatic women. The likelihood of NICU admission was higher amongst neonates from symptomatic mothers with COVID-19. Women with preeclampsia or pregnancy-induced hypertension had a higher likelihood of being symptomatic. Symptomatic pregnant women with COVID-19 also had a greater requirement for ICU admission and mechanical ventilation. There was inadequate data regarding management strategies used for each subgroup due to which differences in management practices could not be compared.

A wide spectrum of disease severity exists amongst pregnant women with COVID-19 disease, with 86% exhibiting mild disease, 9.3% severe, and 4.7% critical [27]. This percentage is similar to those calculated from the non-pregnant adult population (with 80% mild, 15% severe, and 5% critical disease) [28]. However, information regarding the reason for progression to critical disease, depending on the basis of clinical features of COVID-19, is still lacking in the literature.

Previously, the risk of delivery via cesarean section has been reported to be higher amongst pregnant women with COVID-19 disease compared to the general pregnant population [29]. However, whether the presence of symptomatic disease predisposed pregnant women infected with SARS-CoV-2 to a certain mode of delivery is unknown. Our meta-analysis found cesarean section to be more likely amongst symptomatic pregnant women and vaginal delivery to be more likely amongst asymptomatic pregnant women.

A direct link has been established in the literature between SARS-CoV-2 infection and premature labour [30]. A higher rate (37.7%) of preterm birth is seen in pregnant women with COVID-19 as compared to the general pregnant population (12%) [29]. Another systematic review specified that preterm birth before 37 weeks gestation was prevalent in 21.8% of the pregnant women affected by COVID-19 [30]. In terms of assessing the risk of preterm delivery in symptomatic versus asymptomatic pregnant women, our meta-analysis found significant differences between the two groups i.e. higher preterm births in symptomatic pregnant women and similarly, lower birth weight among those infants. Ideally we would have done a subgroup analysis for preterm births but separating the data for term and preterm births was not possible.

Newborns from symptomatic pregnant women were more likely to have lower birthweight. These findings from our review are in line with most studies reporting neonatal outcomes of pregnant women with COVID-19 [27].

The limitations for this review included: (i) a limited number of included studies, (ii) smaller sample size per study, (iii) lack of data on management strategies for both groups, (iv) lack of data on other variables including demographics and co-morbidities from all studies, (v) few studies reporting adjusted analysis and (vi) dearth of evidence from low- and middle-income settings. To obtain conclusive results, more detailed data is required, especially on areas like demographics (co-morbidity, ethnicity, etc.) and maternal outcomes (including those occurring prior to or after COVID-19 diagnosis, e.g. GDM, preeclampsia, etc.). Multivariable analysis to identify factors associated with management, pregnancy, and perinatal outcomes in symptomatic versus asymptomatic pregnant women with COVID-19 could not be done due to insufficient data. However, if data on individual patients is provided in the future, then individual patient data meta-analysis (IPD-MA) would be the ideal approach to provide insights into recognizing and managing COVID-19 disease based on symptoms.

In conclusion, the findings of this study summarize the risk factors, pregnancy, and perinatal outcomes amongst pregnant women based on whether they were symptomatic at presentation. According to this review, obese, hypertensive pregnant women with COVID-19 or those with the respiratory disorder were more likely to be symptomatic. Black or Asian pregnant women with COVID-19 were more likely to be symptomatic while White pregnant women were more likely to be asymptomatic. Delivery via c-section was more likely amongst symptomatic pregnant women while vaginal delivery was more likely amongst asymptomatic pregnant women. Lower mean birthweight was reported among neonates of symptomatic pregnant women and their odds of having LBW babies and preterm births was also higher. Symptomatic pregnant women had a greater requirement for maternal ICU admission and mechanical ventilation and their neonates had a higher likelihood for NICU admission.

The findings of this study, though not very robust can aid to increase the understanding of the course of the disease amongst the two subsets of pregnant women with COVID-19 disease, and its impact on perinatal and neonatal outcomes. This may enable the health care providers to impart better care for the mother and the fetus. However, more studies comparing asymptomatic and symptomatic pregnant women and reporting adjusted pregnancy and birth outcomes are required from varying contexts.

## Supplementary Information

**Additional file 1: Supplementary Table 1**: PRISMA Checklist*.* **Supplementary Table 2**: Search Strategy. **Supplementary Table 3**: Overlapping studies. **Supplementary Table 4**: NHLBI Quality assessment tool for Cohort studies. **Supplementary Table 5**: NHBLI Quality Assessment tool for Case-control studies. Supplementary Table 6: NHLBI Quality assessment tool for case-series. **Figure** **1**: Smoking. **Figure** **2**: Co-morbidity. **Figure** **3**: Obesity. **Figure 4**: Hypertension. **Figure 5**: Cardiovascular disease. **Figure 6**: Respiratory disease. **Figure 7**: Diabetes Mellitus. **Figure 8**: Hypothyroid. **Figure 9**: Cesarean Section. **Figure 10**: Vaginal Delivery. **Figure 11**: Preterm Birth < 37 weeks. **Figure 12**: Preterm Birth < 34 weeks. **Figure 13**: Maternal ICU admission. **Figure 14**: Maternal Mechanical Ventilation. **Figure 15**: NICU admission.

## Data Availability

The datasets used and/or analysed during the current study are available from the corresponding author on reasonable request.
