# Supplementary material for: Differences in pregnancy and perinatal outcomes among symptomatic versus asymptomatic COVID-19-infected pregnant women: a systematic review and meta-analysis
Source: BMC Pregnancy Childbirth. 2021 Dec 1;21:801. doi: 10.1186/s12884-021-04250-1 (PMC8633904; doi:10.1186/s12884-021-04250-1)
Supplement: Supplementary file 1 — Additional file 1: Supplementary Table 1: PRISMA Checklist. Supplementary Table 2: Search Strategy. Supplementary Table 3: Overlapping studies. Supplementary Table 4: NHLBI Quality assessment tool for Cohort studies. Supplementary Table 5: NHBLI Quality Assessment tool for Case-control studies. Supplementary Table 6: NHLBI Quality assessment tool for case-series. Figure 1: Smoking. Figure 2: Co-morbidity. Figure 3: Obesity. Figure 4: Hypertension. Figure 5: Cardiovascular disease. Figure 6: Respiratory disease. Figure 7: Diabetes Mellitus. Figure 8: Hypothyroid. Figure 9: Cesarean Section. Figure 10: Vaginal Delivery. Figure 11: Preterm Birth < 37 weeks. Figure 12: Preterm Birth < 34 weeks. Figure 13: Maternal ICU admission. Figure 14: Maternal Mechanical Ventilation. Figure 15: NICU admission. [file 12884_2021_4250_MOESM1_ESM.docx]

**Supplementary Tables**

**Supplementary Table 1 PRISMA Checklist**

| **Section/topic** | **#** | **Checklist item** | **Reported on page #** |
| --- | --- | --- | --- |
| **TITLE** | | |  |
| Title | 1 | Identify the report as a systematic review, meta-analysis, or both. | 1 |
| **ABSTRACT** | | |  |
| Structured summary | 2 | Provide a structured summary including, as applicable: background; objectives; data sources; study eligibility criteria, participants, and interventions; study appraisal and synthesis methods; results; limitations; conclusions and implications of key findings; systematic review registration number. | 1 |
| **INTRODUCTION** | | |  |
| Rationale | 3 | Describe the rationale for the review in the context of what is already known. | 3 |
| Objectives | 4 | Provide an explicit statement of questions being addressed with reference to participants, interventions, comparisons, outcomes, and study design (PICOS). | 3 |
| **METHODS** | | |  |
| Protocol and registration | 5 | Indicate if a review protocol exists, if and where it can be accessed (e.g., Web address), and, if available, provide registration information including registration number. | 4 |
| Eligibility criteria | 6 | Specify study characteristics (e.g., PICOS, length of follow-up) and report characteristics (e.g., years considered, language, publication status) used as criteria for eligibility, giving rationale. | 4 |
| Information sources | 7 | Describe all information sources (e.g., databases with dates of coverage, contact with study authors to identify additional studies) in the search and date last searched. | 4 |
| Search | 8 | Present full electronic search strategy for at least one database, including any limits used, such that it could be repeated. | 4 |
| Study selection | 9 | State the process for selecting studies (i.e., screening, eligibility, included in systematic review, and, if applicable, included in the meta-analysis). | 4 |
| Data collection process | 10 | Describe method of data extraction from reports (e.g., piloted forms, independently, in duplicate) and any processes for obtaining and confirming data from investigators. | 4 |
| Data items | 11 | List and define all variables for which data were sought (e.g., PICOS, funding sources) and any assumptions and simplifications made. | 4 |
| Risk of bias in individual studies | 12 | Describe methods used for assessing risk of bias of individual studies (including specification of whether this was done at the study or outcome level), and how this information is to be used in any data synthesis. | 4 |
| Summary measures | 13 | State the principal summary measures (e.g., risk ratio, difference in means). | 5 |
| Synthesis of results | 14 | Describe the methods of handling data and combining results of studies, if done, including measures of consistency (e.g., I^2^) for each meta-analysis. | 4, 5 |
| Risk of bias across studies | 15 | Specify any assessment of risk of bias that may affect the cumulative evidence (e.g., publication bias, selective reporting within studies). | 4 |
| Additional analyses | 16 | Describe methods of additional analyses (e.g., sensitivity or subgroup analyses, meta-regression), if done, indicating which were pre-specified. | 4, 5 |
| **RESULTS** |  |  |  |
| Study selection | 17 | Give numbers of studies screened, assessed for eligibility, and included in the review, with reasons for exclusions at each stage, ideally with a flow diagram. | 5, 16 |
| Study characteristics | 18 | For each study, present characteristics for which data were extracted (e.g., study size, PICOS, follow-up period) and provide the citations. | 5, 10-12 |
| Risk of bias within studies | 19 | Present data on risk of bias of each study and, if available, any outcome level assessment (see item 12). | 4 |
| Results of individual studies | 20 | For all outcomes considered (benefits or harms), present, for each study: (a) simple summary data for each intervention group (b) effect estimates and confidence intervals, ideally with a forest plot. | 16, 17 |
| Synthesis of results | 21 | Present results of each meta-analysis done, including confidence intervals and measures of consistency. | 5,6,16,17 |
| Risk of bias across studies | 22 | Present results of any assessment of risk of bias across studies (see Item 15). | 13 |
| Additional analysis | 23 | Give results of additional analyses, if done (e.g., sensitivity or subgroup analyses, meta-regression [see Item 16]). | 5, 6 |
| **DISCUSSION** |  |  |  |
| Summary of evidence | 24 | Summarize the main findings including the strength of evidence for each main outcome; consider their relevance to key groups (e.g., healthcare providers, users, and policy makers). | 7 |
| Limitations | 25 | Discuss limitations at study and outcome level (e.g., risk of bias), and at review-level (e.g., incomplete retrieval of identified research, reporting bias). | 7, 8 |
| Conclusions | 26 | Provide a general interpretation of the results in the context of other evidence, and implications for future research. | 8 |
| **FUNDING** |  |  |  |
| Funding | 27 | Describe sources of funding for the systematic review and other support (e.g., supply of data); role of funders for the systematic review. | 1 |

**Supplementary Table 2: Search Strategy**

| ("Symptomatic pregnant" OR ("symptomatic" AND "pregnant") OR "Pregnant" OR "Woman, pregnant" OR "Pregnant Women"[MeSH Terms] OR "Pregnant Woman" OR "Pregnant Women" OR "Pregnancy" OR "Pregnancies" OR "Pregnant" OR "Gravid" OR "Gravidity" OR "Pregnancy"[MeSH Terms] OR "Gestation" OR "mid-pregnancy" OR "Obstetric" OR "Maternal" OR "Pregnant Females" OR "Parturient")  AND  ("Coronavirus" OR "corona virus" OR "corona virus disease""COVID-19" OR "2019 novel coronavirus disease" OR "COVID19" OR "COVID-19 pandemic" OR "SARS-CoV-2 infection" OR "Severe acute respiratory syndrome coronavirus 2" OR "COVID-19 virus disease" OR "2019 novel coronavirus infection" OR "2019-nCoV infection" OR "coronavirus disease 2019" OR "coronavirus disease-19" OR "2019-nCoV disease" OR "COVID-19 virus infection" OR "corona virus 2019" OR "sars-cov-2" OR "sars-cov2" OR ("wuhan*" AND (("virus" OR "viruses" OR "viral") OR "coronav*")) OR ("covid*" AND ("virus" OR "viruses" OR "viral")) OR "sars-cov-2" OR "sars cov 2" OR "2019nCoV" OR "novel corona virus")  AND  (“asymptomatic” OR “asymptomatic pregnant” OR (“asymptomatic” AND “pregnant”))  AND  (“outcomes” OR “symptoms” OR “Signs and Symptoms”[MeSH Terms] OR “Clinical presentation” OR “Clinical Characteristics” OR “Clinical manifestation*” OR “Laboratory test*” OR “Laboratory characteristics” OR “Complications”[Subheading] OR “Prevalence”[MeSH Terms] OR “Treatment*” OR “Morbidity” OR “Mortality” OR “Clinical features” OR “ventilatory support” OR “Antiviral therapy” OR “corticosteroids” OR “oxygen therapy” OR “Chinese medicine” OR “Antibiotics” OR “immunoglobulin” OR “postpartum outcomes” OR “pregnancy outcomes” OR “neonatal outcomes” OR “Comorbidity”[MeSH Terms]) OR “Prognosis” OR “Management”) |
| --- |

**Supplementary Table 3: Overlapping studies**

| **Overlapping Studies** | | | |
| --- | --- | --- | --- |
| **Study** | **Center, Country and Time period** | **Overlap Study** | **Center, Country and Time period** |
| Dongmei Cao, 2020(1) | Maternal and Child  Health Hospital  Wuhan, China  Jan 23^rd^ to Feb 23^rd^, 2020  Sample size: 10 | Chunchen Wu, 2020(4) | Maternal and Child  Health Hospital  Wuhan, China  January 23 to February 10, 2020  Sample size: 8 |
| Torri Metz, 2020(2) | 33 National Institute of  Child Health and Human Development (NICHD)  Maternal-Fetal Medicine Units (MFMU) sites in 14 states  USA  March 1^st^ to July 31^st^, 2020  Sample size: 1219 | Noelle Breslin, 2020(5) | Columbia University Irving Medical Center (New York, NY) or the Allen Hospital (New York, NY), which are affiliated hospitals of the NewYorkPresbyterian Hospital system  NYC, USA  March 13, 2020, to March 27, 2020  Sample size: 43 |
| Torri Metz, 2020(2) | 33 National Institute of  Child Health and Human Development (NICHD)  Maternal-Fetal Medicine Units (MFMU) sites in 14 states  USA  March 1^st^ to July 31^st^, 2020  Sample size: 1219 | Rasha Khoury, 2020(6) | Five New York City medical centers, including NewYork-Presbyterian Columbia  NYC, USA  March 13^th^ to April 12^th^, 2020  Sample size: 241 |
| Torri Metz, 2020(2) | 33 National Institute of  Child Health and Human Development (NICHD)  Maternal-Fetal Medicine Units (MFMU) sites in 14 states  USA  March 1^st^ to July 31^st^, 2020  Sample size: 1219 | Malavika Prabhu, 2020(7) | 3 New York Presbyterian Hospitals  New York, USA  March 22^nd^ to March 24^th^, 2020  Sample size: 70 |
| Torri Metz, 2020(2) | 33 National Institute of  Child Health and Human Development (NICHD)  Maternal-Fetal Medicine Units (MFMU) sites in 14 states  USA  March 1^st^ to July 31^st^, 2020  Sample size: 1219 | Miranda Delahoy, 2020(8) | COVID-19-Associated Hospitalization Surveillance Network (COVID-NET) from 13 states  USA  March 1^st^ to August 22^nd^, 2020  Sample size: 598 |
| Torri Metz, 2020(2) | 33 National Institute of  Child Health and Human Development (NICHD)  Maternal-Fetal Medicine Units (MFMU) sites in 14 states  USA  March 1^st^ to July 31^st^, 2020  Sample size: 1219 | Allie Sakowicz, 2020(9) | Northwestern Memorial Hospital  Chicago, USA  April 8^th^ to May 31^st^, 2020  Sample size: 101 |
| Nicola Vousden, 2021(3) | UK Obstetric Surveillance 39 System (UKOSS) across all 194 hospitals  UK  March 1^st^ to August 31^st^, 2020  Sample size: 1148 | Asma Khalil, 2020(10) | Portland Hospital for Women and Children, London, UK  March 27^th^ to April 20^th^, 2020  Sample size: 9 |

**Supplementary Table 4: NHLBI Quality assessment tool for Cohort studies**

| **Study ID** | **1. Was the research question or objective in this paper clearly stated?** | **2. Was the study population clearly specified and defined?** | **3. Was the participation rate of eligible persons at least 50%?** | **4. Were all the subjects selected or recruited from the same or similar populations (including the same time period)? Were inclusion and exclusion criteria for being in the study prespecified and applied uniformly to all participants?** | **5. Was a sample size justification, power description, or variance and effect estimates provided?** | **6. For the analyses in this paper, were the exposure(s) of interest measured prior to the outcome(s) being measured?** | **7. Was the timeframe sufficient so that one could reasonably expect to see an association between exposure and outcome if it existed?** | **8. For exposures that can vary in amount or level, did the study examine different levels of the exposure as related to the outcome (e.g., categories of exposure, or exposure measured as continuous variable)?** | **9. Were the exposure measures (independent variables) clearly defined, valid, reliable, and implemented consistently across all study participants?** | **10. Was the exposure(s) assessed more than once over time?** | **11. Were the outcome measures (dependent variables) clearly defined, valid, reliable, and implemented consistently across all study participants?** | **12. Were the outcome assessors blinded to the exposure status of participants?** | **13. Was loss to follow-up after baseline 20% or less?** | **14. Were key potential confounding variables measured and adjusted statistically for their impact on the relationship between exposure(s) and outcome(s)?** |
| --- | --- | --- | --- | --- | --- | --- | --- | --- | --- | --- | --- | --- | --- | --- |
| London 2020 (22) | Yes | Yes | NA | Yes | No | Yes | No | NA | Yes | NA | Yes | NA | NA | No |
| Metz 2021(21) | Yes | Yes | NA | Yes | No | Yes | No | NA | Yes | NA | Yes | NA | NA | Yes |
| Saccone 2020(17) | Yes | Yes | NA | Yes | No | Yes | No | NA | Yes | NA | Yes | NA | NA | Yes |
| Santhosh 2020(18) | Yes | Yes | NA | Yes | No | Yes | No | NA | Yes | NA | Yes | NA | NA | No |
| Verma 2020(20) | Yes | Yes | NA | Yes | No | Yes | No | NA | Yes | NA | Yes | NA | NA | No |
| Vousden 2021(19) | Yes | Yes | NA | Yes | No | Yes | No | NA | Yes | NA | Yes | NA | NA | Yes |

**Supplementary Table 5: NHBLI Quality Assessment tool for Case-control studies**

| **Study ID** | **1. Was the research question or objective in this paper clearly stated?** | **2. Was the study population clearly specified and defined?** | **3. Did the authors include a sample size justification?** | **4. Were controls selected or recruited from the same or similar population that gave rise to the cases (including the same timeframe)?** | **5. Were the definitions, inclusion and exclusion criteria, algorithms or processes used to identify or select cases and controls valid, reliable, and implemented consistently across all study participants?** | **6. Were the cases clearly defined and differentiated from controls?** | **7. If less than 100 percent of eligible cases and/or controls were selected for the study, were the cases and/or controls randomly selected from those eligible?** | **8. Was there use of concurrent controls?** | **9. Were the investigators able to confirm that the exposure/risk occurred prior to the development of the condition or event that defined a participant as a case?** | **10. Were the measures of exposure/risk clearly defined, valid, reliable, and implemented consistently (including the same time period) across all study participants?** | **11. Were the assessors of exposure/risk blinded to the case or control status of participants?** | **12. Were key potential confounding variables measured and adjusted statistically in the analyses? If matching was used, did the investigators account for matching during study analysis?** |
| --- | --- | --- | --- | --- | --- | --- | --- | --- | --- | --- | --- | --- |
| Jenabi 2020 (26) | Yes | Yes | No | Yes | Yes | Yes | NA | No | Yes | Yes | NA | Yes |

**Supplementary Table 6: NHLBI Quality assessment tool for case-series**

| **Study ID** | **1. Was the study question or objective clearly stated?** | **2. Was the study population clearly and fully described, including a case definition?** | **3. Were the cases consecutive?** | **4. Were the subjects comparable?** | **5. Was the intervention clearly described?** | **6. Were the outcome measures clearly defined, valid, reliable, and implemented consistently across all study participants?** | **7. Was the length of follow-up adequate?** | **8. Were the statistical methods well-described?** | **9. Were the results well-described?** |
| --- | --- | --- | --- | --- | --- | --- | --- | --- | --- |
| Hu 2020(25) | No | No | Yes | No | NA | Yes | NA | No | Yes |
| Cao 2020(23) | Yes | Yes | Yes | Yes | NA | Yes | NA | Yes | Yes |
| Wu 2020(24) | Yes | Yes | Yes | Yes | NA | Yes | NA | Yes | Yes |

**Supplementary Figures**

Figure 1 Smoking


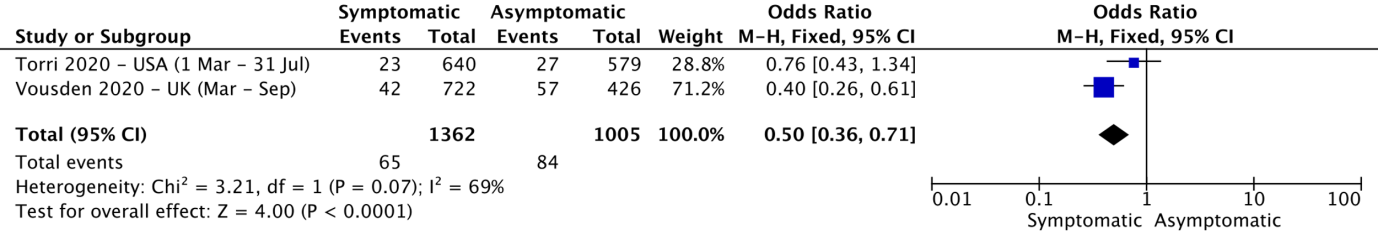


Figure 2 Co-morbidity


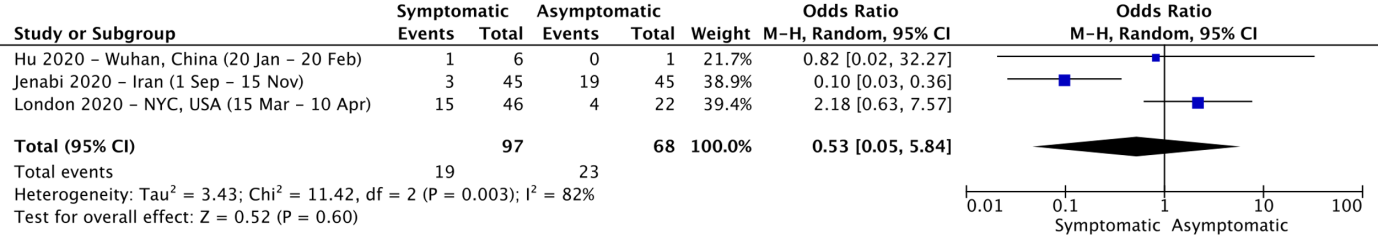


Figure 3 Obesity


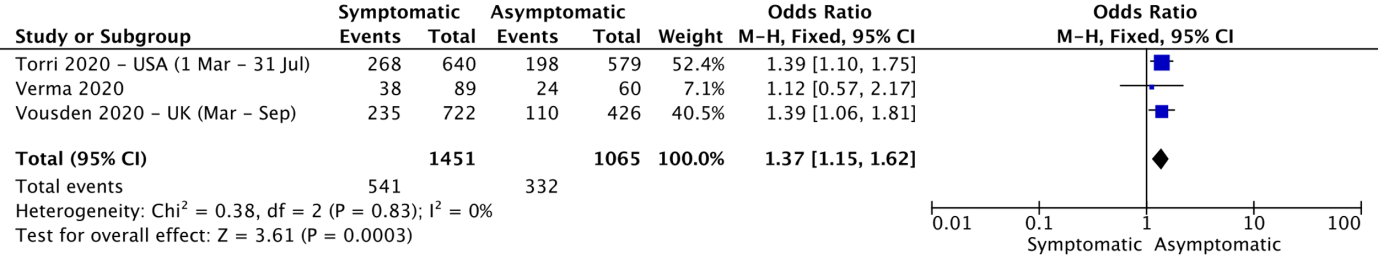


Figure 4 Hypertension


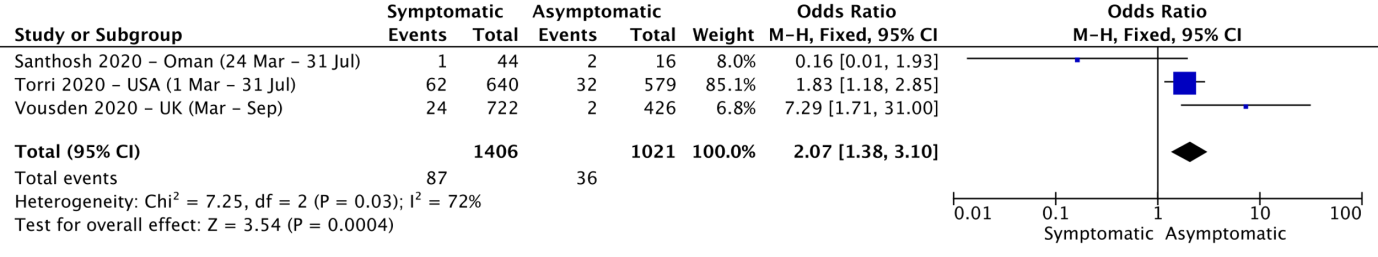


Figure 5 Cardiovascular disease


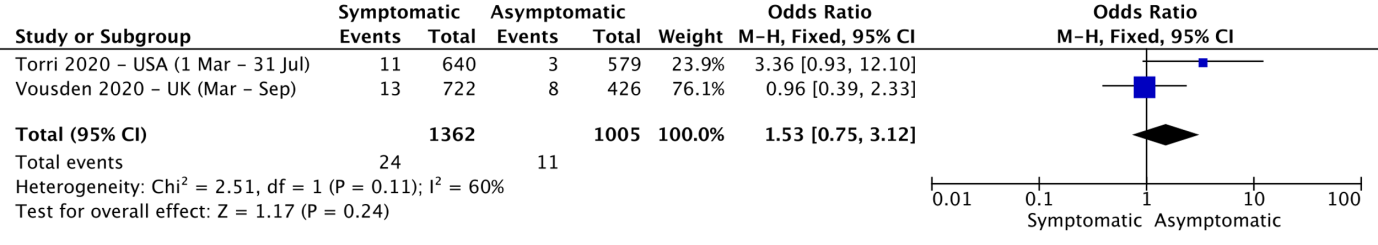


Figure 6 Respiratory disease


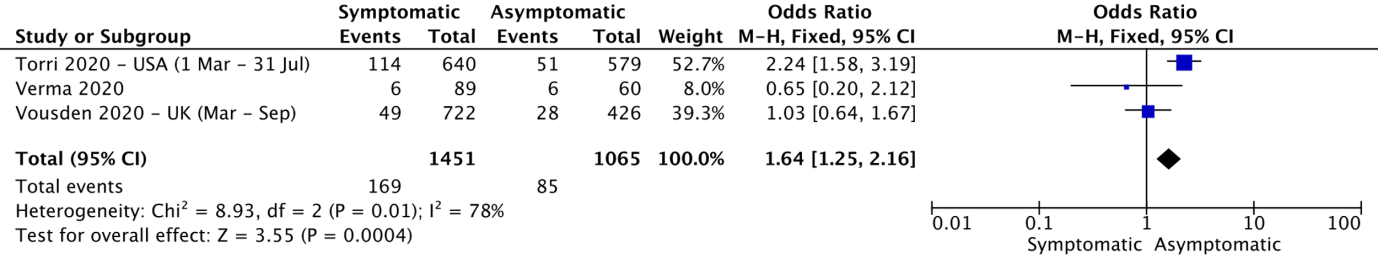


Figure 7 Diabetes Mellitus


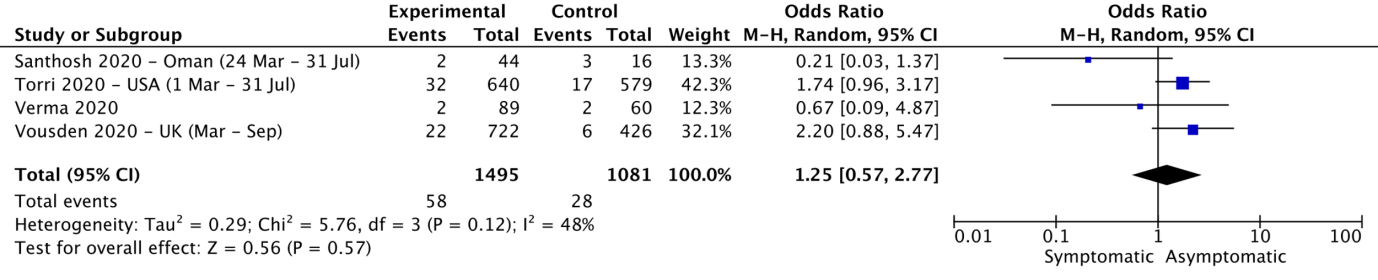


Figure 8 Hypothyroid


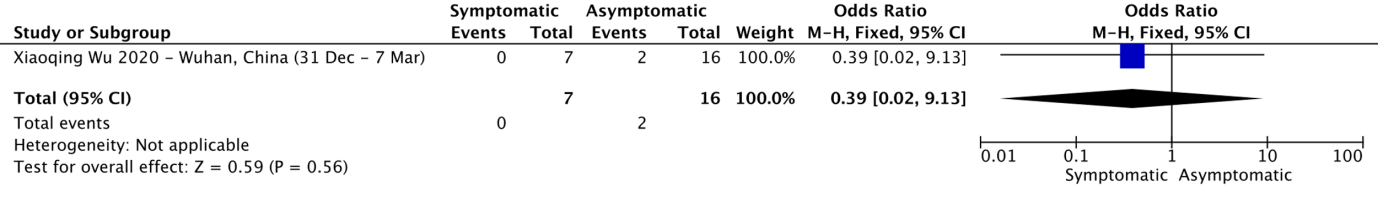


Figure 9 Cesarean Section


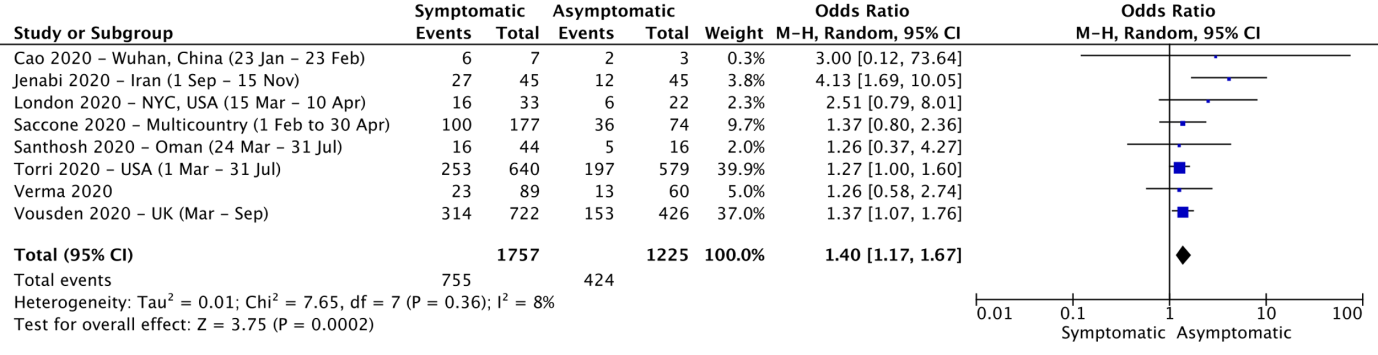


Figure 10 Vaginal Delivery


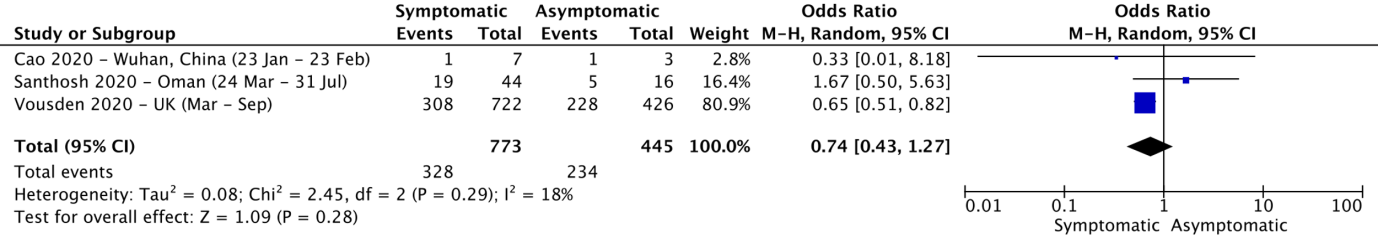


Figure 11 Preterm Birth < 37 weeks


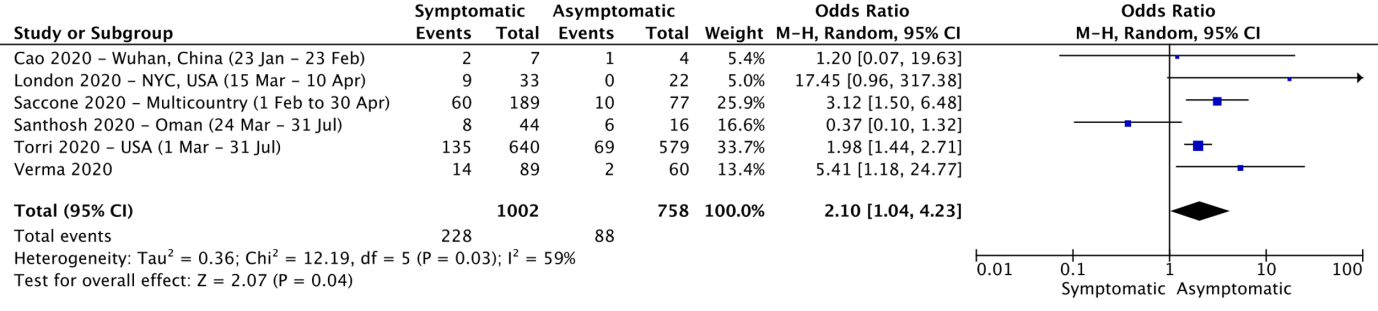


Figure 12 Preterm Birth < 34 weeks


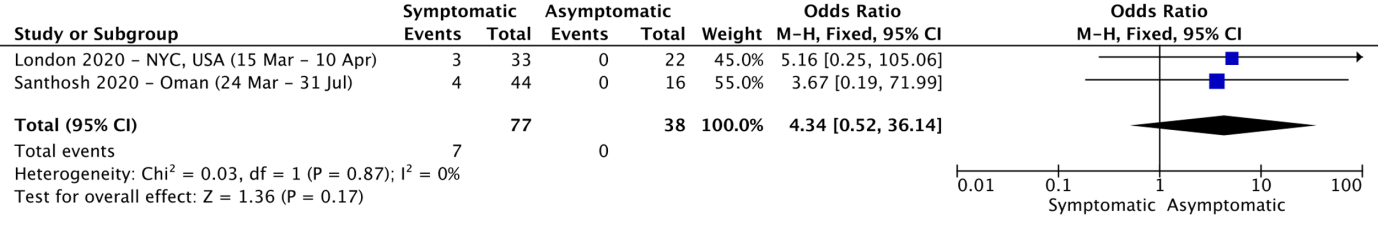


Figure 13 Maternal ICU admission


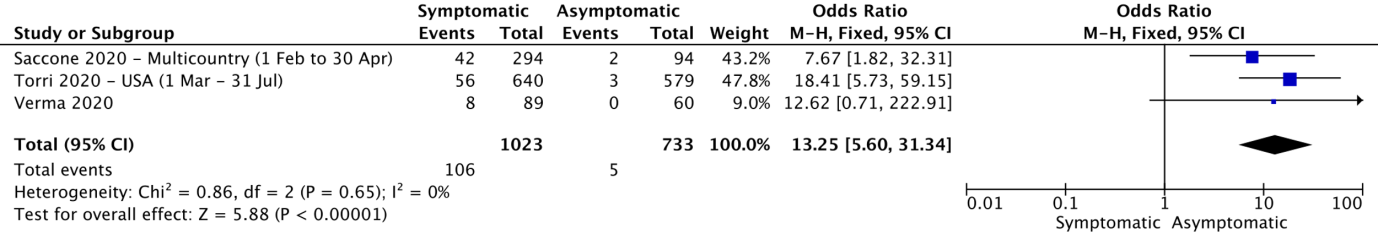


Figure 14 Maternal Mechanical Ventilation


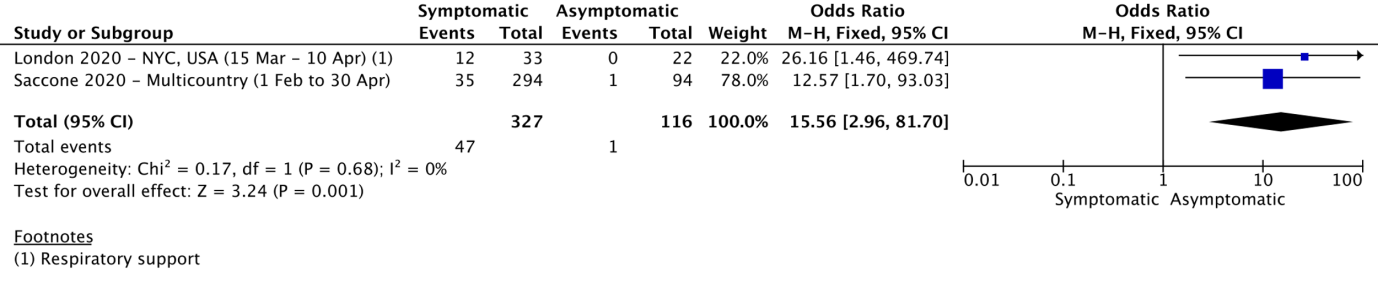


Figure 15 NICU admission


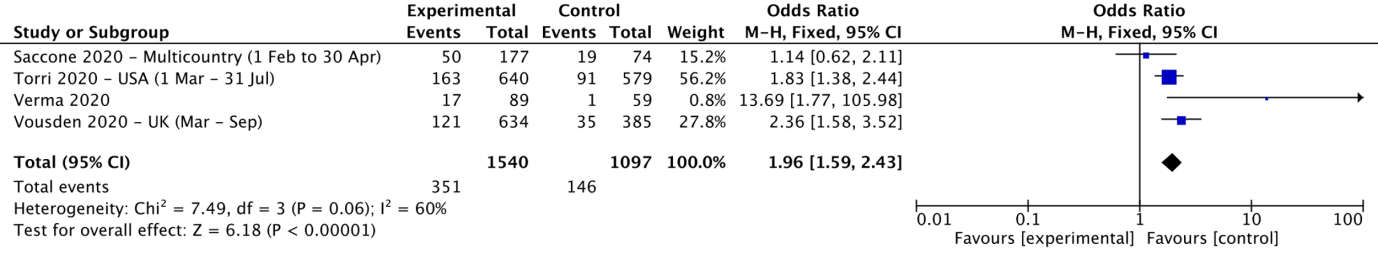


**References:**

1. Cao D, Yin H, Chen J, Tang F, Peng M, Li R, et al. Clinical analysis of ten pregnant women with COVID-19 in Wuhan, China: A retrospective study. Int J Infect Dis. 2020;
2. Metz TD, Clifton RG, Hughes BL, Sandoval G, Saade GR, Grobman WA, et al. Disease Severity and Perinatal Outcomes of Pregnant Patients With Coronavirus Disease 2019 (COVID-19). Obstet Gynecol [Internet]. 2021 Apr;137(4):571–80.
3. Vousden N, Bunch K, Morris E, Simpson N, Gale C, O’Brien P, et al. The incidence, characteristics and outcomes of pregnant women hospitalized with symptomatic and asymptomatic SARS-CoV-2 infection in the UK from March to September 2020: a national cohort study using the UK Obstetric Surveillance System (UKOSS).
4. Wu C, Yang W, Wu X, Zhang T, Zhao Y, Ren W, et al. Clinical Manifestation and Laboratory Characteristics of SARS-CoV-2 Infection in Pregnant Women. Virol Sin. 2020;
5. Breslin N, Baptiste C, … CG-B-A journal of, 2020 undefined. COVID-19 infection among asymptomatic and symptomatic pregnant women: Two weeks of confirmed presentations to an affiliated pair of New York City hospitals. Elsevier.
6. Khoury R, Bernstein PS, Debolt C, Stone J, Sutton DM, Simpson LL, et al. Characteristics and Outcomes of 241 Births to Women With Severe Acute Respiratory Syndrome Coronavirus 2 (SARS-CoV-2) Infection at Five New York City Medical Centers. Obstet Gynecol. 2020;
7. Prabhu M, Cagino K, Matthews KC, Friedlander RL, Glynn SM, Kubiak JM, et al. Pregnancy and postpartum outcomes in a universally tested population for SARS-CoV-2 in New York City: A prospective cohort study. BJOG An Int J Obstet Gynaecol. 2020
8. Delahoy, Miranda J et al. “Characteristics and Maternal and Birth Outcomes of Hospitalized Pregnant Women with Laboratory-Confirmed COVID-19 - COVID-NET, 13 States, March 1-August 22, 2020.” MMWR. Morbidity and mortality weekly report vol. 69,38 1347-1354. 25 Sep. 2020
9. Sakowicz A, Ayala AE, Ukeje CC, Witting CS, Grobman WA, Miller ES. Risk Factors for SARS-CoV2 Infection in Pregnant Women. Am J Obstet Gynecol MFM. 2020
10. Khalil A, Hill R, Ladhani S, Pattisson K, O’Brien P. Severe acute respiratory syndrome coronavirus 2 in pregnancy: symptomatic pregnant women are only the tip of the iceberg. American Journal of Obstetrics and Gynecology. 2020 .
